# Supplementary figures and images for: Proteome and morphological analysis show unexpected differences between promastigotes of Leishmania amazonensis PH8 and LV79 strains
Source: PLoS One. 2022 Aug 23;17(8):e0271492. doi: 10.1371/journal.pone.0271492 (PMC9398010; doi:10.1371/journal.pone.0271492)

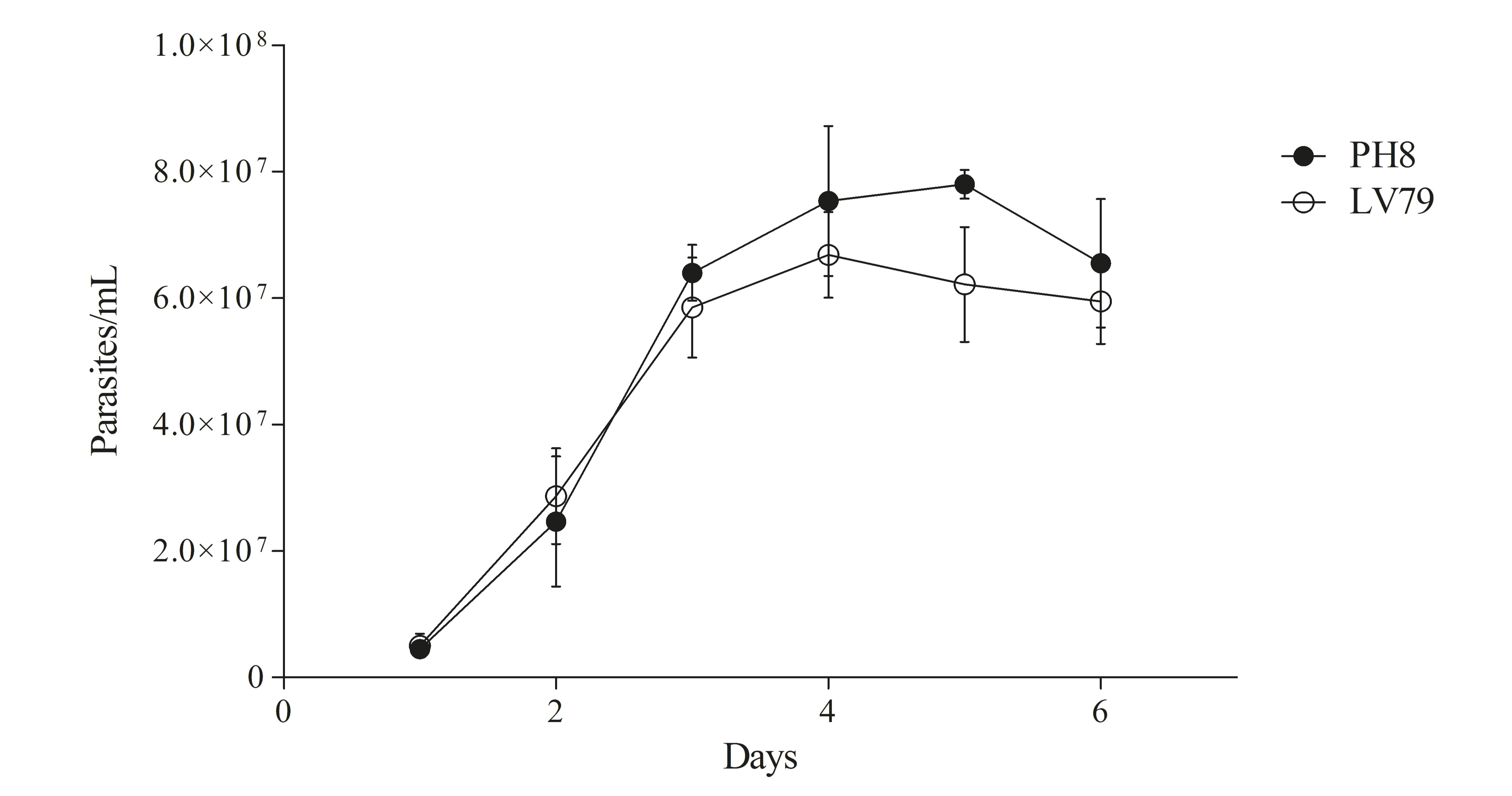

Supplement: S1 Fig — PH8 and LV79 promastigotes were cultured in 199 medium at 24°C and culture density was calculated daily over 6 days. Cultures were initiated with 2 x 10^6^ promastigotes/mL on day 0. Data represented as mean ± SD of three biological replicates. (TIF) [file pone.0271492.s001.tif]

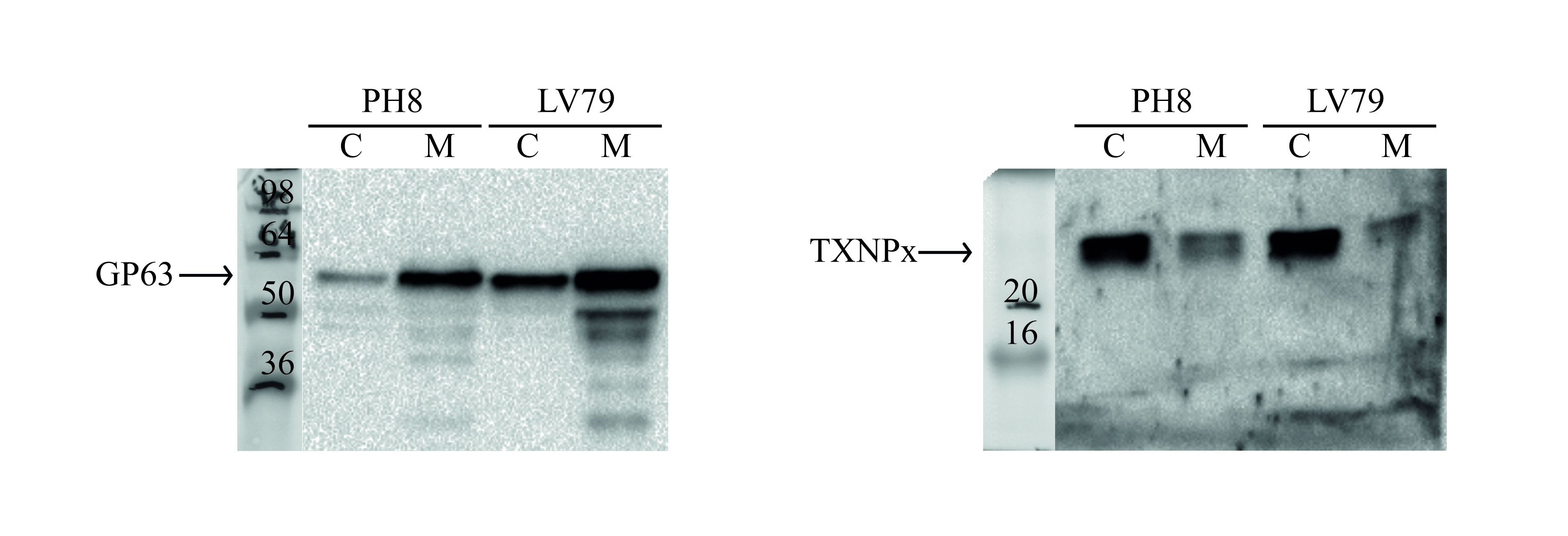

Supplement: S2 Fig — Before proteomics analysis, membrane enrichment was confirmed by Western blot of cytoplasmic (C) and membrane-enriched (M) extracts with anti-GP63 and anti-TXNPx antibodies. Data shown is representative of three analyses performed with different extracts from paired PH8 and LV79 promastigotes cultures. (TIF) [file pone.0271492.s002.tif]

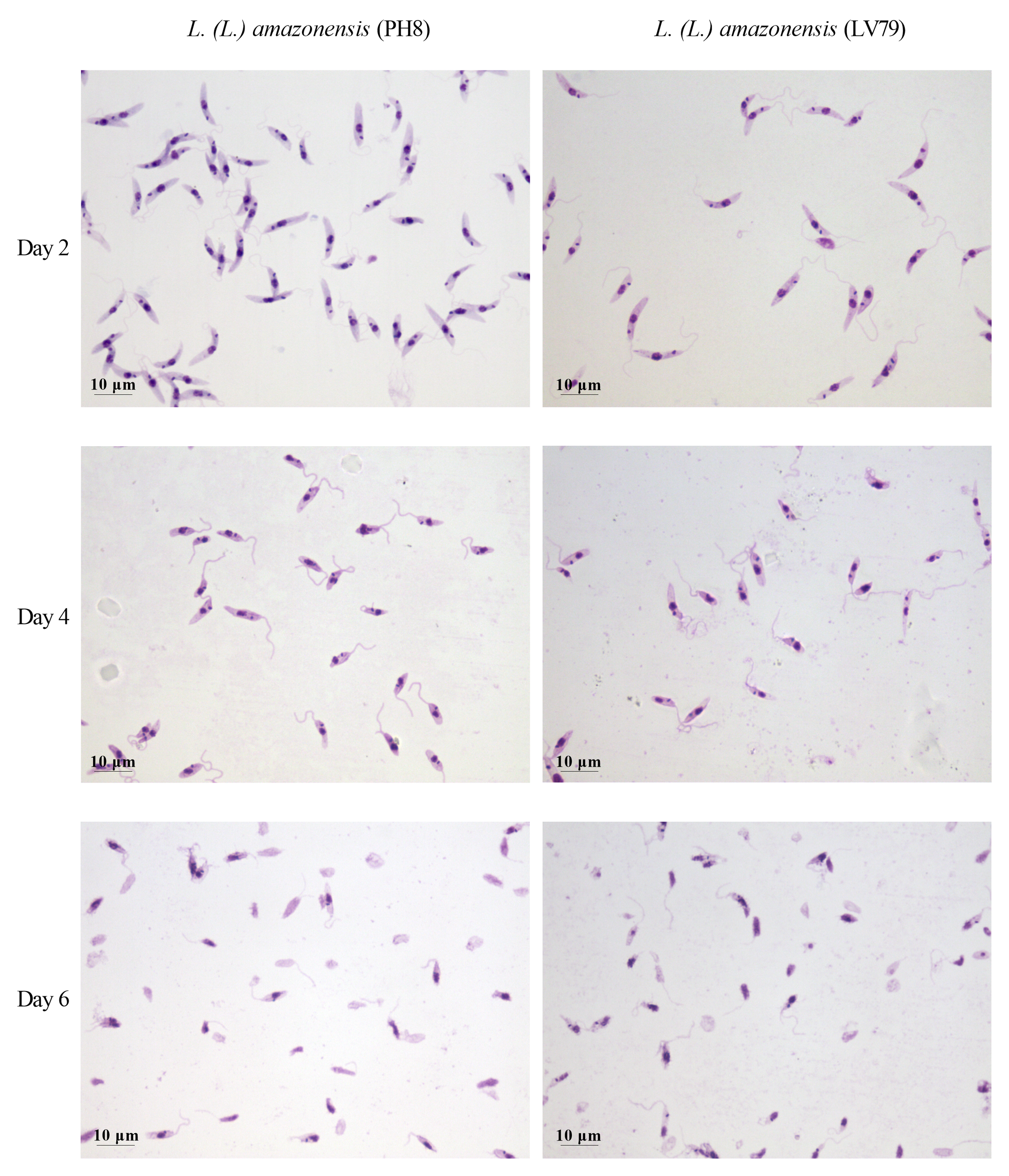

Supplement: S3 Fig — Cultures were synchronized, and parasites were counted, fixed and stained with Giemsa at days 2, 4 and 6 of culture. (TIF) [file pone.0271492.s003.tif]
