# Supplementary material for: Proteome and morphological analysis show unexpected differences between promastigotes of Leishmania amazonensis PH8 and LV79 strains
Source: PLoS One. 2022 Aug 23;17(8):e0271492. doi: 10.1371/journal.pone.0271492 (PMC9398010; doi:10.1371/journal.pone.0271492)
Supplement: S1 Raw images — (PDF) [file pone.0271492.s009.pdf]

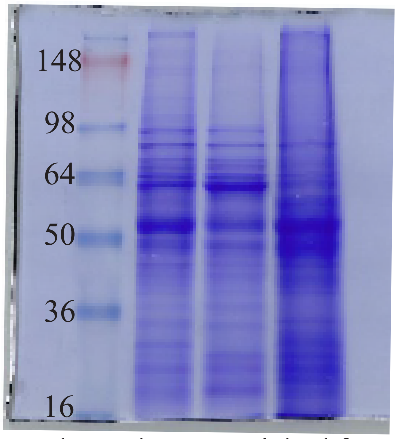

20 µg of protein of total cell, cytoplasmic and membrane-enriched fractions (from left to right) of LV79 promastigotes analyzed by SDS-PAGE stained with Coomassie Brilliant blue R-250. Image from Figure 2A.

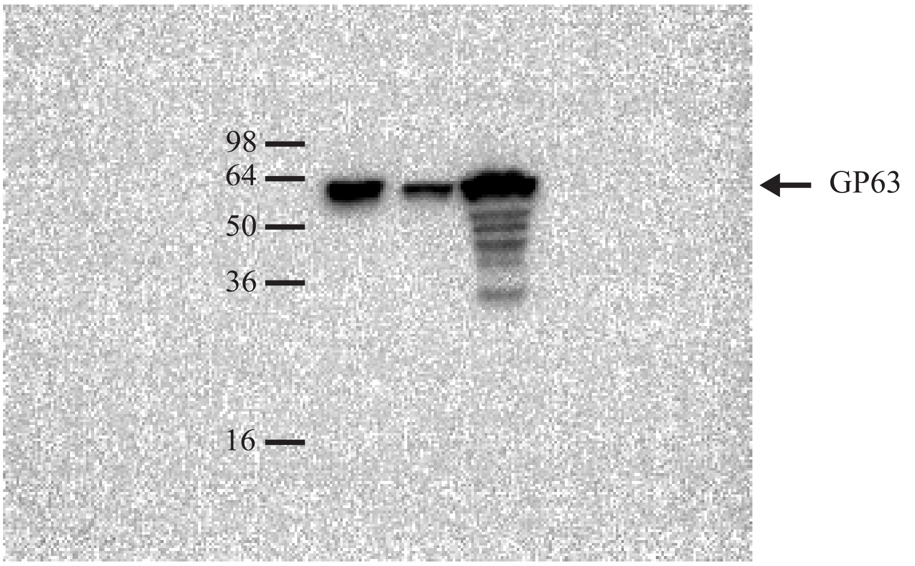

15 µg of protein of total cell, cytoplasmic and membrane-enriched fractions (from left to right) of LV79 promastigotes analyzed by Western blot with anti-GP63 primary antibody and anti-mouse IgG HRP-conjugated secondary antibody. Membranes were incubated with ECL Prime Western Blotting Detection Reagent (GE healthcare) and chemiluminescence was detected by the ChemiDoc XRS+ Imaging system (BioRad). Image from Figure 2B

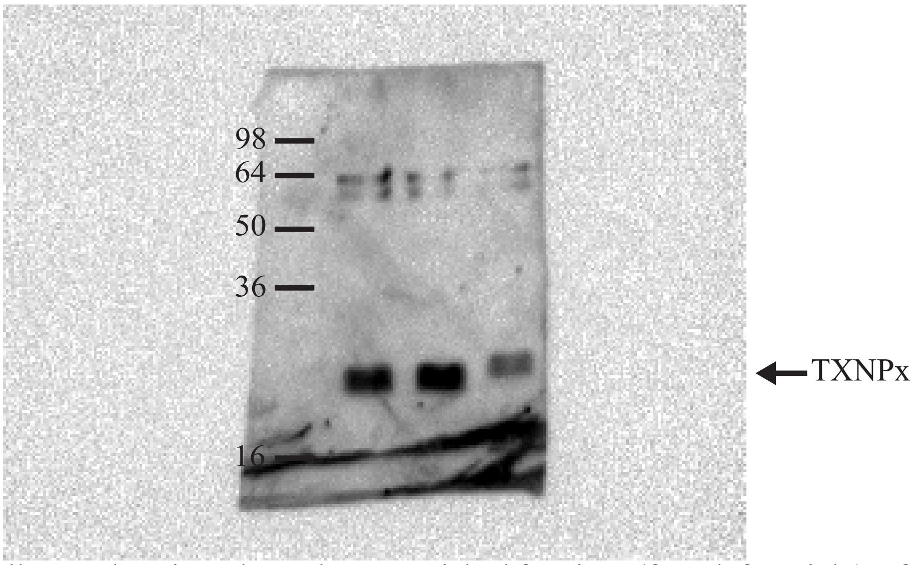

15 µg of protein of total cell, cytoplasmic and membrane-enriched fractions (from left to right) of LV79 promastigotes analyzed by Western blot with anti-TXNPx primary antibody and anti-mouse IgG HRP-conjugated secondary antibody. Membranes were incubated with ECL Prime Western Blotting Detection Reagent (GE healthcare) and chemiluminescence was detected by the ChemiDoc XRS+ Imaging system (BioRad). Image from Figure 2B.

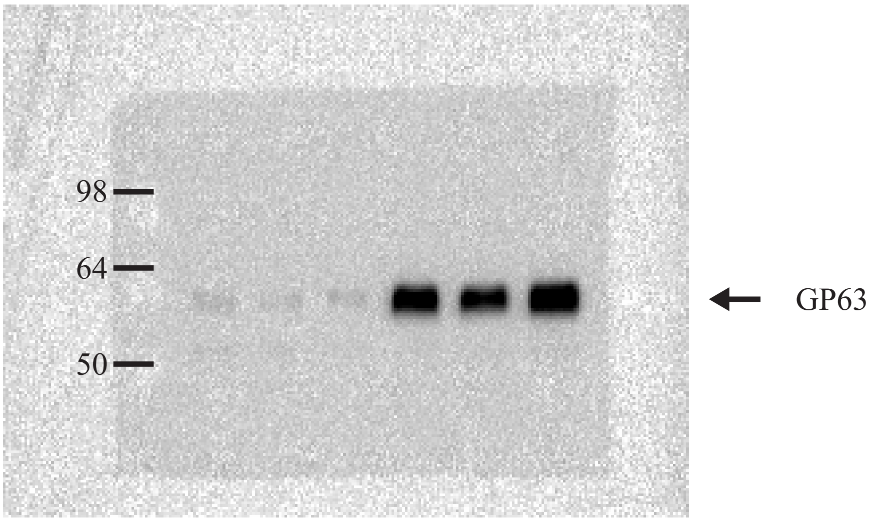

Total cell extracts (15 µg of protein) of PH8 (left) and LV79 (right) promastigotes (three biological samples for each) analyzed by Western blot with anti-GP63 primary antibody and anti-mouse IgG HRP-conjugated secondary antibody. Membranes were incubated with ECL Prime Western Blotting Detection Reagent (GE healthcare) and chemiluminescence was detected by the ChemiDoc XRS+ Imaging system (BioRad). Image from Figure 6B.

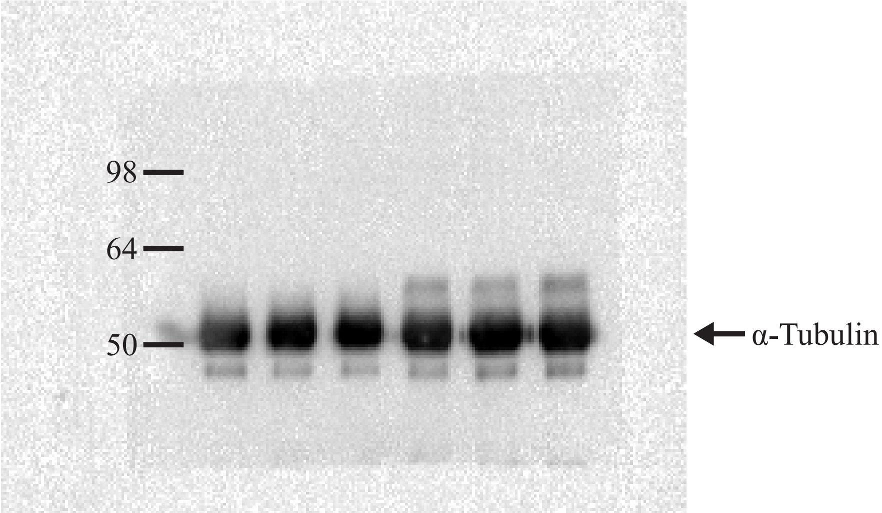

Total cell extracts (15 µg of protein) of PH8 (left) and LV79 (right) promastigotes (three biological samples for each) analyzed by Western blot with anti-α-Tubulin primary antibody and anti-mouse IgG HRP-conjugated secondary antibody. Membranes were incubated with ECL Prime Western Blotting Detection Reagent (GE healthcare) and chemiluminescence was detected by the ChemiDoc XRS+ Imaging system (BioRad). Image from Figure 6B.

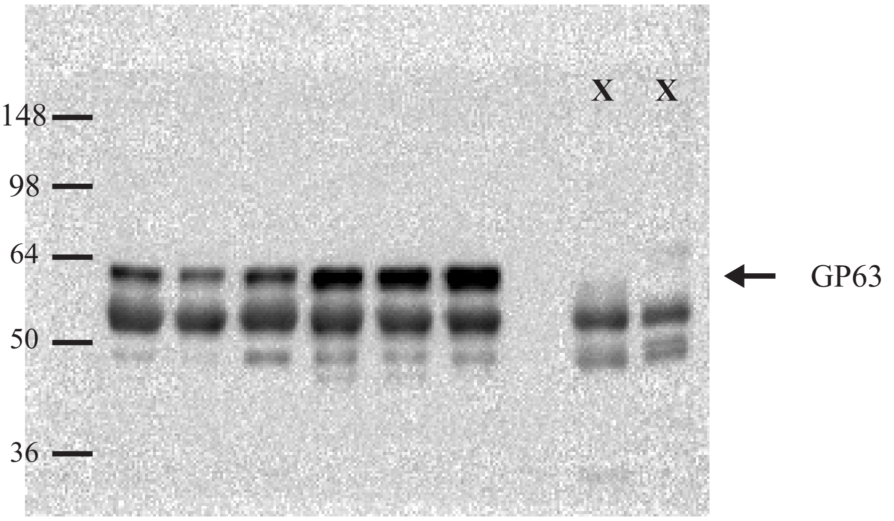

Membrane enriched fractions (15 µg of protein) of PH8 (left) and LV79 (right) promastigotes (three biological samples for each) analyzed by Western blot with anti-GP63 primary antibody and anti-mouse IgG HRP-conjugated secondary antibody. Membranes were incubated with ECL Prime Western Blotting Detection Reagent (GE healthcare) and chemiluminescence was detected by the ChemiDoc XRS+ Imaging system (BioRad). Image from Figure 6B.
X - unrelated samples.

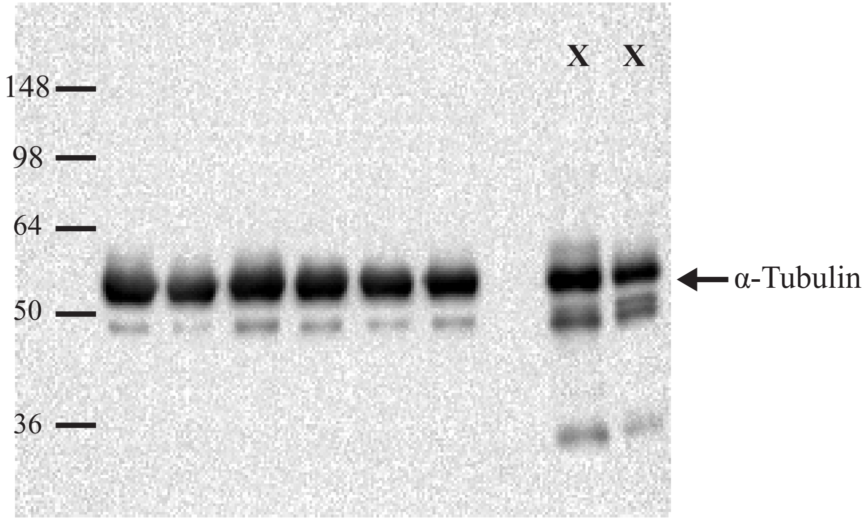

Membrane enriched fractions (15 µg of protein) of PH8 (left) and LV79 (right) promastigotes (three biological samples for each) analyzed by Western blot with anti-α-Tubulin primary antibody and anti-mouse IgG HRP-conjugated secondary antibody. Membranes were incubated with ECL Prime Western Blotting Detection Reagent (GE healthcare) and chemiluminescence was detected by the ChemiDoc XRS+ Imaging system (BioRad). Image from Figure 6B. X - unrelated samples.

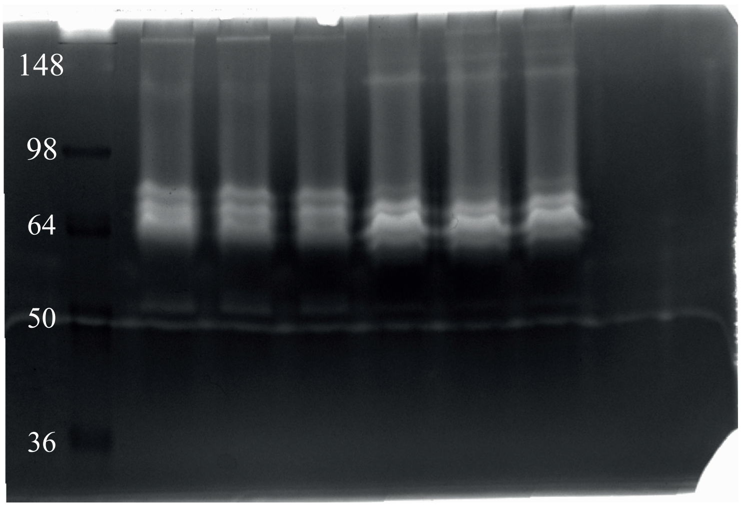

Total cell extracts (2 µg of protein) of PH8 (left) and LV79 (right) promastigotes (three biological samples for each) analyzed by zymography. Image from Figure 6C.

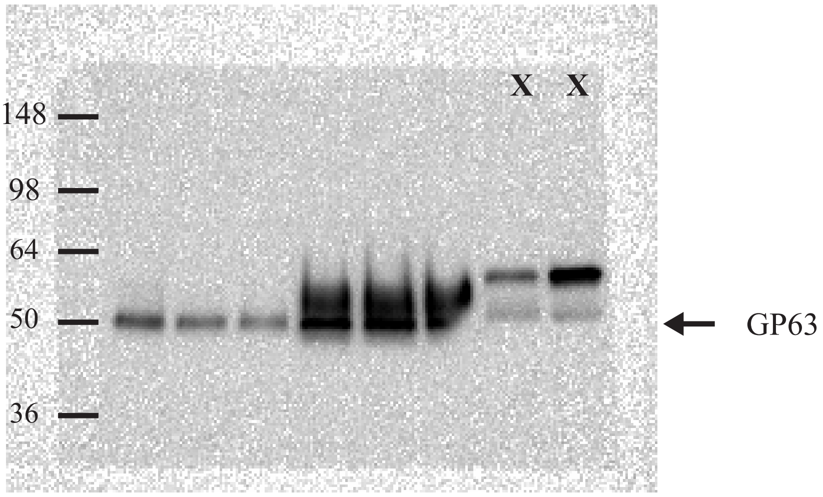

Total cell extracts (15 µg of protein) of PH8 (left) and LV79 (right) promastigotes (three biological samples for each) analyzed by non-reducing Western blot with anti-α-GP63 primary antibody and anti-mouse IgG HRP-conjugated secondary antibody. Membranes were incubated with ECL Prime Western Blotting Detection Reagent (GE healthcare) and chemiluminescence was detected by the ChemiDoc XRS+ Imaging system (BioRad). Image from Figure 6C. X - unrelated samples.

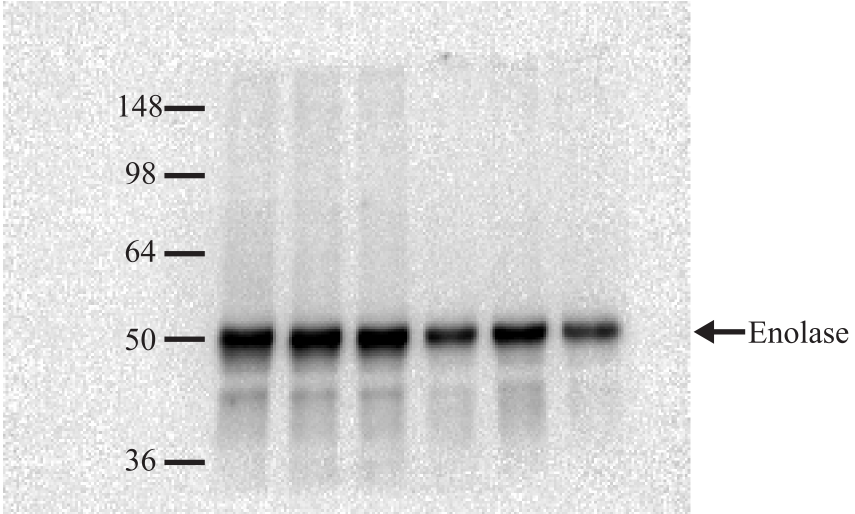

Total cell extracts (15 µg of protein) of PH8 (left) and LV79 (right) promastigotes (three biological samples for each) analyzed by Western blot with anti-enolase primary antibody and anti-mouse IgG HRP-conjugated secondary antibody. Membranes were incubated with ECL Prime Western Blotting Detection Reagent (GE healthcare) and chemiluminescence was detected by the ChemiDoc XRS+ Imaging system (BioRad). Image from Figure 7B.

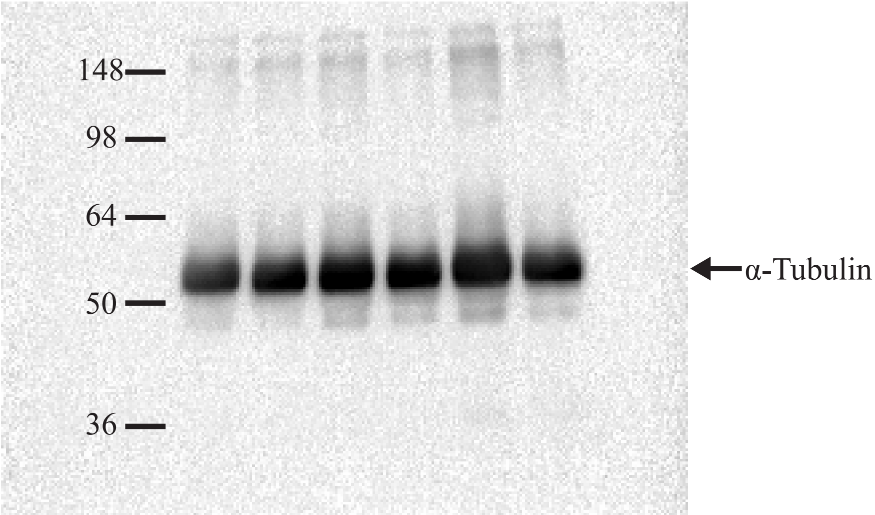

Total cell extracts (15 µg of protein) of PH8 (left) and LV79 (right) promastigotes (three biological samples for each strain) analyzed by Western blot with anti-α-Tubulin primary antibody and anti-mouse IgG HRP-conjugated secondary antibody. Membranes were incubated with ECL Prime Western Blotting Detection Reagent (GE healthcare) and chemiluminescence was detected by the ChemiDoc XRS+ Imaging system (BioRad). Image from Figure 7B.

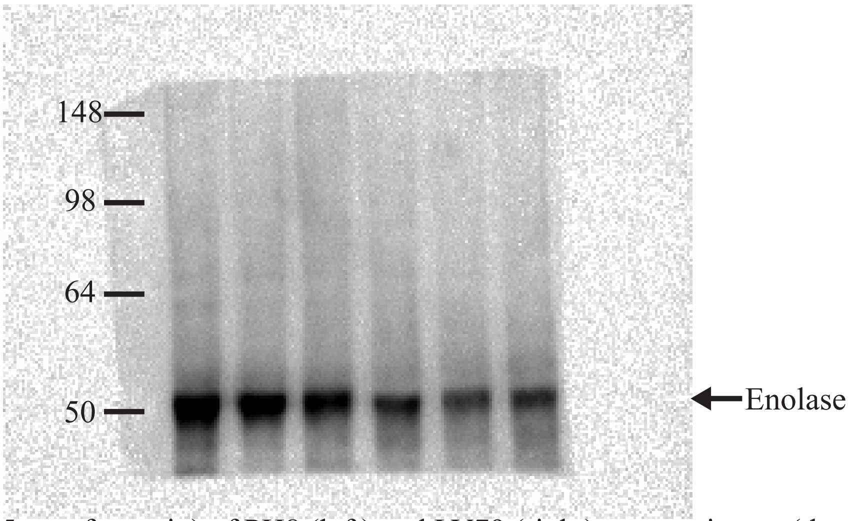

Membrane enriched fractions (15 µg of protein) of PH8 (left) and LV79 (right) promastigotes (three biological samples for each) analyzed by Western blot with anti-enolase primary antibody and anti-mouse IgG HRP-conjugated secondary antibody. Membranes were incubated with ECL Prime Western Blotting Detection Reagent (GE healthcare) and chemiluminescence was detected by the ChemiDoc XRS+ Imaging system (BioRad). Image from Figure 7B.

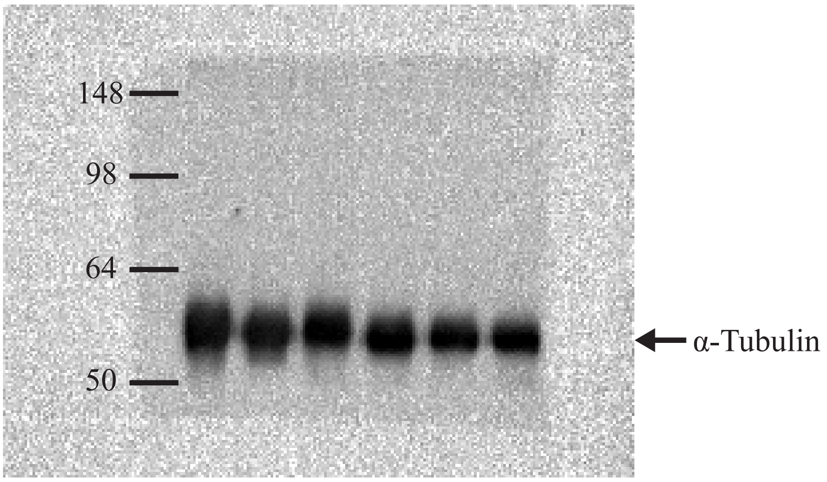

Membrane enriched fractions (15 µg of protein) of PH8 (left) and LV79 (right) promastigotes (three biological samples for each) analyzed by Western blot with anti-α-Tubulin primary antibody and anti-mouse IgG HRP-conjugated secondary antibody. Membranes were incubated with ECL Prime Western Blotting Detection Reagent (GE healthcare) and chemiluminescence was detected by the ChemiDoc XRS+ Imaging system (BioRad). Image from Figure 7B.

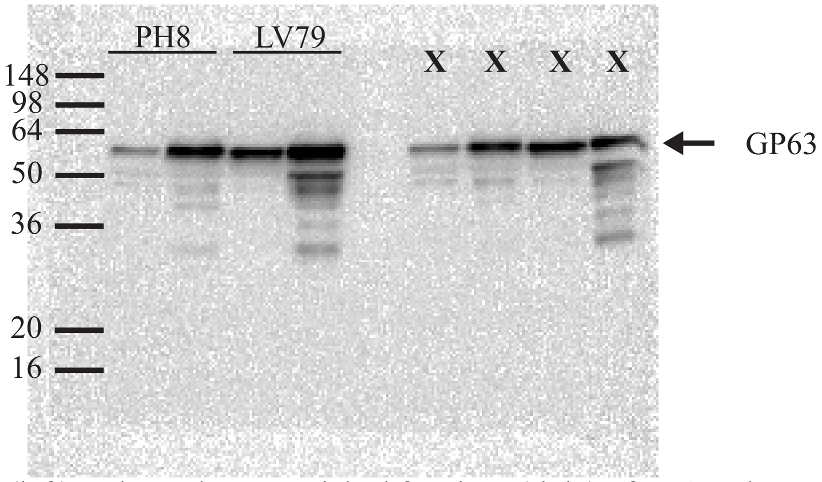

15 µg of protein of cytoplasmic (left) and membrane-enriched fractions (right) of PH8 and LV79 promastigotes and analyzed by Western blot with anti-GP63 primary antibody and anti-mouse IgG HRP-conjugated secondary antibody. Membranes were incubated with ECL Prime Western Blotting Detection Reagent (GE healthcare) and chemiluminescence was detected by the ChemiDoc XRS+ Imaging system (BioRad). Image from Supplementary Figure 2. X - unrelated samples.

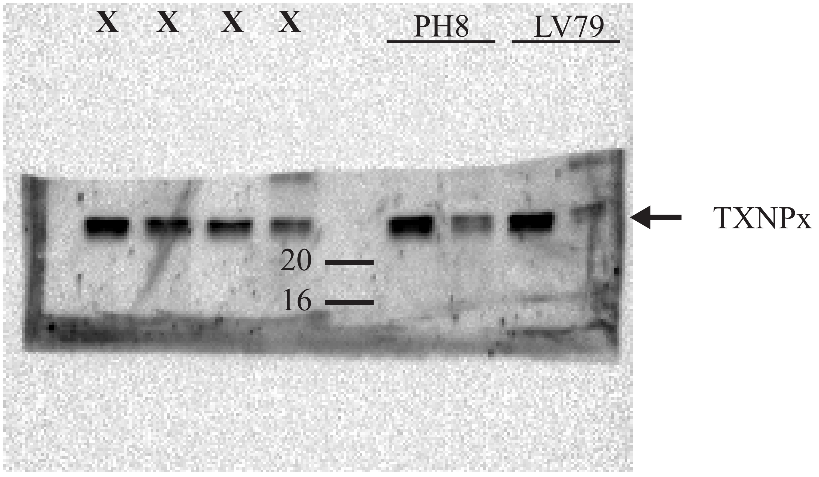

15 µg of protein of cytoplasmic (left) and membrane-enriched fractions (right) of PH8 and LV79 promastigotes and analyzed by Western blot with anti-TXNPx primary antibody and anti-mouse IgG HRP-conjugated secondary antibody. Membranes were incubated with ECL Prime Western Blotting Detection Reagent (GE healthcare) and chemiluminescence was detected by the ChemiDoc XRS+ Imaging system (BioRad). Image from Supplementary Figure 2. X - unrelated samples.
