# Supplementary material for: Proteome and morphological analysis show unexpected differences between promastigotes of Leishmania amazonensis PH8 and LV79 strains
Source: PLoS One. 2022 Aug 23;17(8):e0271492. doi: 10.1371/journal.pone.0271492 (PMC9398010; doi:10.1371/journal.pone.0271492)
Supplement: Attachment — Submitted filename: Reviewers Complete Reply.pdf [file pone.0271492.s010.pdf]

RESPONSE TO REVIEWERS

We would like to thank the reviewers and editors for the careful revision and for the possibility of improving our manuscript for publication in PlosOne.

Please find below the answers for the questions PNTD posed to reviewers and for the questions/comments the reviewers sent to us on a separate file.

1) PNTD- Reviewer's Responses to Questions

Key Review Criteria Required for Acceptance?
As you describe the new analyses required for acceptance, please consider the following:

Methods
-Are the objectives of the study clearly articulated with a clear testable hypothesis stated?
-Is the study design appropriate to address the stated objectives?
-Is the population clearly described and appropriate for the hypothesis being tested?
-Is the sample size sufficient to ensure adequate power to address the hypothesis being tested?
-Were correct statistical analysis used to support conclusions?
-Are there concerns about ethical or regulatory requirements being met?

Reviewer #2: (No Response)

Reviewer #3: - Yes, for the most part
- not entirely
- no
- yes
- yes
- no

Does the analysis presented match the analysis plan?
-Are the results clearly and completely presented?
-Are the figures (Tables, Images) of sufficient quality for clarity?

Reviewer #2: (No Response)

Reviewer #3: - yes
- yes, for the most part. Authors should improve: information/units on graph axis in the figures not only in captions, the bioinformatics analyses performed (GO annotation and other bioinformatic prediction tools), and other paragraphs highlighted in the submitted notes.
- The figures embedded in the pdf file are blurry and some values are difficult to read (see notes).

R: Thank you for the careful revision and requests. I believe we have addressed all issues. Figure quality is limited by file size, we improved as much as possible and hope it is now

readable.

Conclusions
-Are the conclusions supported by the data presented?
-Are the limitations of analysis clearly described?
-Do the authors discuss how these data can be helpful to advance our understanding of the topic under study?
-Is public health relevance addressed?

Reviewer #2: (No Response)

Reviewer #3: - to some extent. Contradictory information remains to be explained.
- yes
- to some extent. A study focussing on membrane-enriched fractions should expand on exosome/vesicle-based secretion which is extremely important for Leishmania pathogenesis but it is hardly mentioned.
- yes

R: We agree with the reviewer that some contradictory results were obtained when we compared proteomic profile with infectivity and culture composition. Proteomic analysis of PH8 strain, the most virulent and infective one, indicated higher abundance of proteins commonly related to metacyclics, while morphological/morphometrical analysis showed that LV79 culture has a higher percentage of metacyclics and molecular markers showed no differences between strains. It is possible that proteins different from the ones reported for other species characterize metacyclics in *L. amazonensis*, and/or that markers used for other species cannot be used for this one. Further studies by us and other groups are necessary to fully address these points.

Anyway, infectivity in vivo (previous study) and in vitro (this study) have similar patterns: PH8 promastigotes (day 4 cultures) infect more macrophages and generates larger lesions in mice than LV79.

We do agree that exosome and vesicles play very important roles in *Leishmania* infection. Although our proteome analysis probably doesn’t include shed exosomes due to several parasite washing steps, many proteins detected in our proteome were decribed in exosomes, as is the case of GP63 and enolase. Exosomes are now mentioned in the discussion about gp63 and in perspectives. Thank you for the observation.

Editorial and Data Presentation Modifications?
Use this section for editorial suggestions as well as relatively minor modifications of existing data that would enhance clarity. If the only modifications needed are minor and/or editorial, you may wish to recommend “Minor Revision” or “Accept”.

Reviewer #2: (No Response)

Reviewer #3: a) Material and methods:

– the origin/supplier of some reagents is not indicated (e.g. BSA, anti-Leishmania serum, Bradford assay, antibodies) or incorrect (ProlongTM Diamond, Waters Corporation). Inconsistencies in showing suppliers' location.

R: Thank you. We have revised suppliers' information.

- Real time PCR – primer sequences should indicate 5'- ... – 3' as well as forward or reverse.
- "RT-PCR or qPCR reactions were performed on a StepOne …". Also, "DDCt" should be ether DeltaDelta or use the proper Greek symbol.

R: Thank you. We have revised all issues mentioned.

b) Fig 2 (and Supp Fig 2) – images for GP63 membranes seem to be very altered (contrast) and TXNP membranes with a lot of background…
R: Images were not altered; they are display as obtained from the equipment ChemiDoc XRS (BioRad).

c) Supplementary Table 1 – What information is in line 2? Also, empty lines 271/273…

R: Sorry. We have revised that.

d) Supplementary tables 2 and 3 – Need caption or title. LFQ values should be shown as this is the main quantitative data used in the manuscript.

R: Thank you. The requested information is provided in the revised Sup Tables 2 and 3.

We also send a revised Sup Table 4 in which we include mean LFQ intensity values used for fold calculation.

e) Caption Fig 3 - "B. Clustering of the six samples (three biological samples (S1, S2 and S3) for each strain) by principal component analysis of all proteins identified."
In graph, samples are named P2, P4, P7.

R: Thank you. We have modified sample names in the revised Fig3.

f) S1 table – columns BR and BS should be between columns U and V.

Summary and General Comments
Use this section to provide overall comments, discuss strengths/weaknesses of the study, novelty, significance, general execution and scholarship. You may also include additional comments for the author, including concerns about dual publication, research ethics, or publication ethics. If requesting major revision, please articulate the new experiments that are needed.

Reviewer #2: (No Response)

Reviewer #3: The manuscript submitted by Tano et al. analyses two strains of Leishmania amazonensis (PH8 and LV79), causing different clinical presentations of cutaneous leishmaniases in mice, as shown in their previous publication (reference 15). In this study, the

authors analysed adhesion and phagocytosis by bone marrow-derived macrophages and performed a proteomic analysis of day 4 promastigote membrane-enriched fractions. In the manuscript, authors show that the PH8 strain adheres more and is phagocytosed at higher rates than the LV79 strain, and several differentially expressed proteins are identified, using LFQ quantification. The manuscript also includes a bioinformatic analysis (GO annotation and other prediction algorithms) to hint on protein function and possibly explain the differences observed in pathogenesis.
The experimental validation of the proteomic data is robust and very thorough. Authors performed several complementary techniques to assess metacyclogenesis and the presence of important virulence factors. Yet, many contradictory data remain unexplained particularly regarding the differences in observed in vivo virulence of both strains. One possible solution could be a more in-depth analysis of the most abundant differentially expressed proteins as well as of identified uncharacterised proteins. Also, the lack of culture viability data is a significant limitation of the presented data which could also possibly explain some of the observed contradictions.
Overall, the manuscript is well written, although the overall English quality could be improved in some sections (namely discussion). The manuscript should demonstrate more clearly how the study's results relate to existing knowledge, particularly, more meaningful comparisons with previous proteomic study on soluble amastigote fractions of these two strains could be provided.

R: We thank this reviewer for the positive opinion concerning many aspects of our work. The reviewer states that "Yet, many contradictory data remain unexplained particularly regarding the differences in observed in vivo virulence of both strains". However, infectivity in vivo and in vitro have similar patterns: PH8 promastigotes (day 4 cultures) infect more macrophages and generates larger lesions in mice than LV79. In fact, the higher virulence observed for PH8 (in vivo) prompted us to compare phagocytosis, and the higher binding and internalization of this strains stimulated the search for differential proteins.

----

2) Questions posed by reviewers on a separate file

1. In Abstract and introduction:

"Leishmaniases are diseases caused by Leishmania protozoans that affect around 12 million people" / " Around 12 million people have leishmaniasis [1]"
Affect and infect have completely different meanings. In reference 1 there is no mention of leishmaniasis prevalence nor of "12 million people" value.
If authors mean to mention the number of people at risk of developing leishmaniasis (affected by) this number should be 1 billion people (population of 98 endemic countries), as stated in the provided reference 2. Regarding people infected by leishmaniasis, prevalence data is for the most part unavailable due to underreporting (only 25-45% reported cases per year), highly variable access to medical treatment and healing rates, so authors could just mention incidence rates (as they correctly do). I recommend updating this information and corresponding reference. There is more recent data reported in the WHO Weekly

Epidemiological Record number 35, 96, 401-420 (3 september 2021). This last reference also updates the number of endemic countries for VL (89) and CL (79).

R: We thank the referee for the suggestions. We have modified the text accordingly.

2. "Leishmania infantum, Leishmania infantum chagasi and Leishmania donovani are the most frequent etiologic agents of VL".

Leishmania infantum and *Leishmania chagasi* are synonyms. Please see the provided reference 6 and Akhoundi et al (PMID: 26937644).

R: We agree in this point and have modified the text.

3. "In Brazil, seven dermotropic species are associated with TL, and Leishmania braziliensis, Leishmania amazonensis and Leishmania guyanensis are the most frequent ones [6]."

Authors could provide some information on the most common clinical presentations caused by L. amazonensis, the subject of their study.

R: Thank you for the suggestion. We have added a short text mentioning clinical presentations frequently observed in symptomatic *L. amazonensis* infections: "*Leishmania amazonensis* symptomatic infections usually lead to cutaneous localized leishmaniasis, characterized by one or a few ulcerated lesions, and more rarely to the diffuse form, typified by many non-ulcerated lesions with high parasite loads"

4. "Another important membrane-anchored virulence factor is metalloproteinase GP63 [13]."

No state-of-the-art information on the importance of GP63 in pathogenesis is provided.

R: We again agree with the reviewer. We have added the sentence "…, a protein involved in phagocytosis, resistance to complement lysis and subversion of macrophage signaling and activation" and included recent references about GP63 roles in macrophage activation and pathogenesis.

5. "… less robust techniques (such as 2-DE) that do not allow the analysis of a high number of proteins."

While this technique does present some limitations (in the detection of low abundance proteins, extremely acidic or basic and of very high or very low molecular weight), they are still 'robust' techniques.

R: We have modified the text.

6. Please revise sentence to improve clarity "The abundances of all proteins and of the differential ones precisely clustered samples from the same strain [15]."

R: Thank you. We´ve modified this sentence to "A clustering strategy based on the abundance of all proteins identified, as well as a clustering based only on the differential ones…"

7. Please clarify the sentence “Classification of differentially abundant proteins by function suggests that proteomic profile of PH8 includes more traits of metacyclics than LV79 profile.” What do the authors mean with “traits of metacyclics”?

R: By “traits of metacyclics” we mean proteins related to functions commonly associated to metacyclics. We have modified this sentence.

8. “2.2 L. amazonensis promastigotes culture”

Authors do not provide any information on parasite concentration and, more importantly, viability at the moment of collection. Images shown in Figure S3 may indicate significant levels of cell death at day 6. I believe this information to be key to be able to accept subsequent analyses and conclusions based on these samples.

R: Culture densities on day 4 were statistically similar between strains, varying from 6 to 7.35 $x10^7$/mL for LV79 and from 6.6 to 8.8 $x10^7$/mL for PH8, as shown in the growth curve displayed in Sup Fig 1 (curves were calculated from three cultures for each strain).
We haven´t determined viability at the moment of collection. However, Figure S3 shows that very few parasites didn´t seem viable on day 4, differently from day 6 (as also noted by the reviewer), supporting our choice of day 4 cultures.

9. “Cells stained with 0.04% trypan blue were counted..”

This is likely a mistake – 0,4% trypan blue?

R: Sorry, we should have written 0,4%! Thank you very much for such a careful revision.

10. “Protein abundance was calculated based on its normalized spectral intensity (LFQ Intensity).”

Authors used LFQ for fold-change calculations but S2 and S3 tables shows iBAQ absolute values. Authors should revise this information and either provide both or keep consistency. Also, according to the MaxQuant definition, LFQ (Label-free quantification) intensities are based on the (raw) intensities and normalised on multiple levels to ensure that profiles of LFQ intensities across samples accurately reflect the relative amounts of the proteins. On the other hand, iBAQ (Intensity-Based Absolute Quantification) values calculated are the (raw) intensities divided by the number of theoretical peptides. Thus, iBAQ values are proportional to the molar quantities of the proteins. Label-free quantification using the iBAQ algorithm provides an accurate description of protein relative abundance and a rough estimate of the molar quantity of a given protein, allowing to conclude on the biological relevance of identified proteins. Why did authors opt for LFQ quantification?

R: Thank you for the suggestion. We have added LFQ values to revised S2 and S3 tables. We have also changed iBAQ values for the mean value of the triplicates for each strain.

11. Not sure what authors mean by “… and LV79 day 2 mean values as references”.

R: GAPDH was used as reference gene, and LV79 day 2 mean values were employed for normalization of all types of samples, so that all values are relative quantifications compared to LV79 day 2. We have modified the sentence in the manuscript to “2−DDCt equation was

employed to calculate the relative expression of each gene in all samples using GAPDH as reference gene. LV79 day 2 mean values were used for normalization of all samples". We have also changed figure legend accordingly.

12. Fig 1 – graph should show clearly values on y -axis, not only in caption. And authors should add exemplary microscopy images to clearly show adhered/phagocytosed promastigotes.

R: We have modified y axis mentioning that 500 macrophages were analyzed for attached and phagocytosed parasites. A revised Fig1 is provided.

13. "Considering the proteins identified in both strains, 62 proteins were identified as more abundant in PH8 samples, while 41 were identified as more abundant in LV79, as shown in the heat map (Figure 3C)."

Four proteins show >10-fold increase in abundance, including Surface-antigen-like proteins and calcium-binding proteins which could be important players in strain-specific pathogenesis. Yet these were not mentioned or discussed anywhere in the manuscript.

R: We agree with the referee that these four proteins could have been mentioned. We have only mentioned the most differential one, LABCG1, in the discussion. We now included the following sentence: "Four proteins showed fold increases above 10 in PH8: Putative ATP-binding cassette protein subfamily G, member 1 (LABCG1), Glutamate dehydrogenase, Putative flagellar calcium-binding protein and Surface antigen-like protein. These proteins may eventually contribute to the increased infectivity of PH8."

14. Figure 4 – it is not clear what information comes from GO annotation and other bioinformatic prediction tools. The image has very low resolution and it is hard to read. Also, 4A shows 42% cytosolic proteins and 4B shows 86% membrane proteins… Results should be presented more clearly and these contradictions should be assessed. Also, to effectively compare between two lists GO enrichment analysis should be performed instead of just GO annotation analysis.

R: The origin of the information is shown in a new table, Sup Table 5. This table shows all proteins identified and their classifications according to GO, DeepLoc, WolfPsort (animal and fungi) and LocTree3. As can be noted, only 588 proteins from the 1659 had GO. For the proteins with no GO classification, a consensus was obtained from the other 3 tools.
Concerning Fig 4, we believe there was a misunderstanding in data interpretation. Graph 4A show 42% of cytosolic proteins, while graph 4B show 86% soluble proteins with no transmembrane domains. We have run a recent analysis of the proteins using the same tools and criteria and the values have changed a little. We thus provide a current version of Fig 4 (Figure 4 rev) and modified the text with current percentages.
Since GO classification was available for only 35,4% of the proteins, we opted not to analyze GO enrichment.

15. Only 3% membrane-associated proteins is extremely low for a study that proposes to analyse membrane-enriched fractions. While authors discuss this issue in the discussion section, the provided references states "below 40%" or 6,5% (Wolf PSort+DeepLoc) with Wolf Psort algorithm alone predicting 17,4%, and the latter still

performed Percoll purifications also on L. amazonensis promastigotes. Perhaps, since many technical difficulties are found in the purification of membrane-enriched fractions, the soluble fractions should be analysed in parallel and used as a negative filter to exclude commonly identified proteins, ensuring that identified proteins are indeed associated exclusively to membrane-enriched fractions instead of concluding on the basis of prediction algorithms. Also, these algorithms present several limitations which could explain the difference between experimental and prediction data, which could be discussed.

R: As noted by the referee and mentioned by us in the discussion, there are certainly limitations on membrane purification using sodium carbonate. However, despite the low enrichment on membrane proteins, GP63 and enolase were validated in membrane and total extracts, suggesting that the data obtained is solid.
It would be interesting to perform simultaneous proteomic analysis of soluble fractions. Unfortunately, we didn´t think about that before having access to the data showing low enrichment, and we´re not able to do that at this moment due to financial constraints.

16. Stating GP63 is more abundant in the LV79 strain with only 0,26 fold change is misleading since there is a negligible level of overexpression. Also, 3 different isoforms of GP63 are detected in all 3 replicates (E9AN53, E9AN57 and E9AZL8) which are not mentioned or discussed. Why were they excluded? And perhaps different isoforms are detected differently by the used antibodies which may explain differences in detection in western blot (Fig 6B) but with no impact on strain pathogenicity or enzyme activity (6C).
    Regarding this, in the discussion section, authors state "Curiously, according to proteomics analysis GP63 was more abundant in membrane-enriched fractions of LV79, which was confirmed by western blot." This sentence and discussion should be revised.

R: We believe that 0,26-fold change may correspond to significant biological effects, since it indicates that LV79 samples have almost 4 times more protein than PH8. We could have mentioned that 3 isoforms of gp63 were detected in all samples. We have focused on E9AN53 because it was the only with differential abundance between the two strains, but now we mention the three isoforms in the revised version of the manuscript: "Three isoforms (E9AN53, E9AN57 and E9AZL8) of GP63, a well-known virulence factor of *Leishmania*, were detected in all samples analyzed. One of these isoforms was more abundant in LV79 membrane-enriched promastigotes fractions…"
The antibody used for GP63 was gently provided by Prof Rob McMaster, and was obtained after immunization of mice with *Leishmania* GP63.
We stated "Curiously, according to proteomics analysis GP63 was more abundant in membrane-enriched fractions of LV79" because we expected that the most virulent strain would express more GP63.

17. Fig 7 – describes LFQ intensities yet the values in the chart correspond likely to iBAQ or absolute intensities.

R: In Fig 7, as well as in Fig 6, we show "raw" (non-log transformed) LFQ values (before log2 conversion). We have added this information to the corresponding legends of the two figures. Please find below other representatins of the same graphs using log2LFQ and iBAQ.

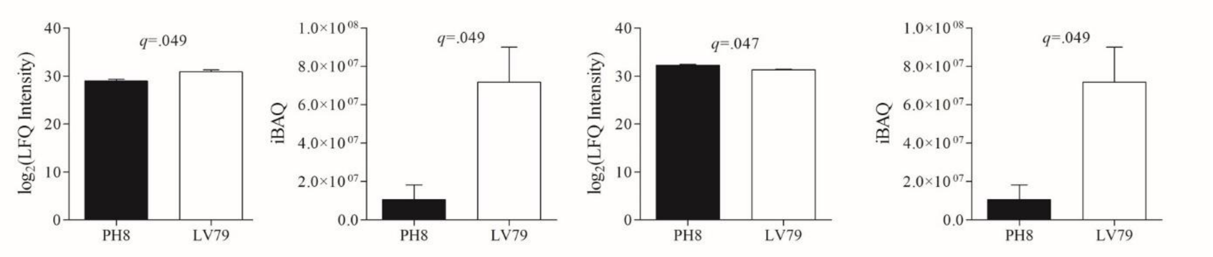

18. "**Proteomic analysis** showed that the majority of the proteins upregulated in LV79 promastigotes participate in translation, while most of the proteins upregulated in PH8 are involved in carbohydrate metabolism, cytoskeleton composition and vesicle and membrane trafficking." – do the authors mean "**GO annotation analysis** showed…"?

R: Indeed, we mean GO annotation analysis! Thank you for the correction, we have modified that in the manuscript.

19. "Studies comparing procyclic and metacyclic promastigotes of L. infantum, L. tropica and L. major revealed that during metacyclogenesis proteins related to translation are downregulated and proteins related to motility and carbohydrate metabolism, upregulated [36-38]. Although there are no similar studies for L. amazonensis, our results suggest that PH8 promastigotes, different from LV79, have a profile that resembles the infective metacyclic form." – This paragraph is a repetition and should only be in the discussion section, as it is already in the paragraph "Several studies have compared procyclic and metacyclic promastigotes of different Leishmania species [36-38]…."

R: We absolutely agree with the reviewer. This paragraph was removed. Thank you very much.

20. Regarding the flow cytometry analysis to determine metacyclic promastigotes, authors declare calculating the frequencies of metacyclic parasites according to reference 16, yet, in this study, parasites we not fixed and PI staining was used to assess viability. Day 5 metacyclic parasites were then gated on FSC/PI- cells (viable). Here, only FSC/SSC were used and the difference between the left panels and right panels in Figure 8A is not clear. In Figure 8A, the left panels show 4,21% and 9,41% of all parasites – were these used to gate the metacyclic parasites on the right panel? If so, there is an incredible amount of debris again suggesting the overall viability of the cultures is very low.
    Low culture viability is argued further when authors state "Data from day 6 was not considered valid due to the huge proportion of parasites without nucleus, without flagellum or with atypical morphology." Also, the 2 sentences before should be removed as they are only describing the material and methods.

R: We apologize for the confusion made by the percentages shown in the left panels of Fig 8A. FSC x SSC plots were performed to separate *Leishmania* from debris (small products generated from culture, solutions and equipment). The gates shown in the left panels represent Leishmania, either dead or alive, and should not have been quantified. The “meaningless” numbers (4,21% and 9,41%) were removed from the revised figure 8. Although we didn´t employ PI labeling to remove dead parasites, the ungated graphs were quite similar to those obtained in reference 16, according to Sandra Muxel, coauthor of both studies.
Regarding the morphometrical analysis, the sentences describing the procedure were removed from the results. Thank you.

21. Regarding the morphometric analysis of promastigotes, in reference 17, day 7 L. major WT promastigotes were assessed and 0% procyclic and 70% metacylic promastigotes were found. These values are clearly very different from the ones found in this study. While different Leishmania species were being studied, I wonder if this information further argues against the quality of the used cultures and using day 4 promastigote cultures to assess metacyclic promastigotes. In addition, the low amount of metacyclic promastigores observed in Figure 9 could also be explained simply by the fact that day 4 promastigotes were used, before metacyclogenesis was completed. In their previous study (reference 15) day 5 promastigotes were used to infect mice. Perhaps differences between strains could be explained by faster and not higher rates of metacyclogenesis. Furthermore, in the discussion section authors state “The higher abundance of metacyclics in LV79 according to the morphological analyses was quite unexpected in face of the higher infectivity of PH8 cultures and the higher similarity of these cultures to metacyclic metabolic profile.” This is perhaps misleading since the overall number of metacyclics in both cultures is very low, and lower than should be expected.

R: We normally use *L. amazonensis* day 4 cultures for phagocytosis and infection assays, since the cultures have already reached stationary phase and has low numbers of dead parasites. Since day 4 cultures of LV79 and PH8 displayed similar densities (Sup Fig1) but differences in binding to macrophages and phagocytosis, we chose them for proteomic analysis. It is indeed probable that the choice of day 4 is one of the factors responsible for the lower proportion of metacyclics compared to *L. major* day 7 culture (ref 17). Besides, there may be differences due to species and strain peculiarities.
In our previous paper describing mouse infection by LV79 and PH8 we used cultures from the same day. At that time, we named the day of subculture as day 1, while we now call it day 0, so the previous day 5 corresponds to the present day 4. Indeed, the growth curve shown in ref 15 is quite similar to the one shown as Sup Fig 1. We apologize for this confusion.
We agree that the number of metacyclics is quite low for both strains, and probably doesn´t affect infectivity of the cultures. We have removed this sentence from the discussion.

22. “However, it is also evident that LV79 cultures at days 2 and 4 show higher proportion of metacyclics compared to PH8 (**8.12 x 0% for day 2, 16.56 x 3.07% for day 4**).” – Authors should revise how they present these result values more clearly.

R: We have rewritten the sentence: “Besides, it is also evident that LV79 cultures at days 2 and 4 show higher proportion of metacyclics compared to PH8 In fact, day 2 cultures of LV79

display 8.12% metacyclics compared to 0% for PH8, and day 4 cultures of LV79 show 16.56% of metacyclics compared to 3.07% for PH8.

23. “These findings are curious but not unexpected, since enzymes may be closely associated to membranes of cell or organelles and may also be kept inside vesicles formed during membrane rupture.” – Authors never mention the importance of exosomes and vesicle-based secretion which are proven to be extremely important in Leishmania pathogenesis, as shown in reference 13 (Atayde et al) and others (to cite a few: i) Silverman, J. M.; Reiner, N. E. Leishmania Exosomes Deliver Preemptive Strikes to Create an Environment Permissive for Early Infection. Front. Cell. Infect. Microbiol. 2012, 1 (January), 1–8. https://doi.org/10.3389/fcimb.2011.00026 /ii) Silverman, J. M.; Clos, J.; Horakova, E.; Wang, A. Y.; Wiesgigl, M.; Kelly, I.; Lynn, M. A.; McMaster, W. R.; Foster, L. J.; Levings, M. K.; Reiner, N. E. Leishmania Exosomes Modulate Innate and Adaptive Immune Responses through Effects on Monocytes and Dendritic Cells. J. Immunol. 2010, 185 (9), 5011–5022. https://doi.org/10.4049/jimmunol.1000541 / iii) Silverman, J. M.; Chan, S. K.; Robinson, D. P.; Dwyer, D. M.; Nandan, D.; Foster, L. J.; Reiner, N. E. Proteomic Analysis of the Secretome of Leishmania Donovani. Genome Biol. 2008, 9 (2), R35. https://doi.org/10.1186/gb-2008-9-2-r35 / iv) Cuervo, P.; De Jesus, J. B.; Saboia-Vahia, L.; Mendonça-Lima, L.; Domont, G. B.; Cupolillo, E. Proteomic Characterization of the Released/Secreted Proteins of Leishmania (Viannia) Braziliensis Promastigotes. J. Proteomics 2009, 73 (1), 79–92. https://doi.org/10.1016/j.jprot.2009.08.006 / v) Silverman, J. M.; Clos, J.; De’Oliveira, C. C.; Shirvani, O.; Fang, Y.; Wang, C.; Foster, L. J.; Reiner, N. E. An Exosome-Based Secretion Pathway Is Responsible for Protein Export from Leishmania and Communication with Macrophages. J. Cell Sci. 2010, 123 (6), 842–852. https://doi.org/10.1242/jcs.056465. / vi) Hassani, K.; Olivier, M. Immunomodulatory Impact of Leishmania-Induced Macrophage Exosomes: A Comparative Proteomic and Functional Analysis. PLoS Negl. Trop. Dis. 2013, 7 (5), e2185. https://doi.org/10.1371/journal.pntd.0002185. / vii) Veras, P. S. T.; De Menezes, J. P. B. Using Proteomics to Understand How Leishmania Parasites Survive inside the Host and Establish Infection. Int. J. Mol. Sci. 2016, 17 (8). https://doi.org/10.3390/ijms17081270 / viii) Rodríguez-Vega, A.; Losada-Barragán, M.; Berbert, L. R.; Mesquita-Rodrigues, C.; Bombaça, A. C. S.; Menna-Barreto, R.; Aquino, P.; Carvalho, P. C.; Padrón, G.; de Jesus, J. B.; Cuervo, P. Quantitative Analysis of Proteins Secreted by Leishmania (Viannia) Braziliensis Strains Associated to Distinct Clinical Manifestations of American Tegumentary Leishmaniasis. *J. Proteomics* **2021**, *232* (December 2020). https://doi.org/10.1016/j.jprot.2020.104077).
Several proteins have been described as exosome markers in Leishmania, even though they were typically considered as cytosolic proteins. These references should be discussed further and may provide further explanation as to why some proteins were identified in the membrane-associated fraction analysed in this study.

R: We do agree with the reviewer that exosome and vesicles play very important roles in *Leishmania* infection, including in *L. amazonensis* experimental model of infection. Although our proteome analysis probably doesn’t include shed exosomes due to several parasite washing steps, many proteins detected in our list are known to be present in exosomes, as is the case of GP63 and enolase. Further studies of exosomes shed by PH8 and LV79

promastigotes would certainly improve our knowledge about pathogenicity of these strains and parasite-host cell communication.

Exosomes are now mentioned in the discussion when we mention gp63 and in perspectives.
